# Effects of yeast culture on in vitro ruminal fermentation and microbial community of high concentrate diet in sheep

**DOI:** 10.1186/s13568-024-01692-6

**Published:** 2024-04-15

**Authors:** Hongze Wang, Guiqiong Liu, Aimin Zhou, Huiguo Yang, Kun Kang, Sohail Ahmed, Biao Li, Umar Farooq, Fuqing Hou, Chaoli Wang, Xue Bai, Yan Chen, Yi Ding, Xunping Jiang

**Affiliations:** 1 Key Laboratory of Agricultural Animal Genetics, Breeding and Reproduction of Ministry of Education, Huazhong Agricultural University, Wuhan, 430070 China; 2 National key Laboratory for Exploitation and Utilization of Agricultural Microbial Resources, Yichang, 443003 China; 3 Institute of Animal Husbandry, Xinjiang Academy of Animal Sciences, Urumqi, 830013 China; 4 National Sheep Industry Technology System Shihezi Comprehensive Experimental Station, Shihezi, 832000 China; 5 Mianyang Academy of Agricultural Sciences, Mianyang, 621023 China

**Keywords:** Yeast culture, Rumen fermentation, Gas production, Bacterial community, Fungal community

## Abstract

This research aimed to investigate effects of different yeast culture (YC) levels on in vitro fermentation characteristics and bacterial and fungal community under high concentrate diet. A total of 5 groups were included in the experiment: control group without YC (CON), YC1 (0.5% YC proportion of substrate dry matter), YC2 (1%), YC3 (1.5%) and YC4 (2%). After 48 h of fermentation, the incubation fluids and residues were collected to analyze the ruminal fermentation parameters and bacterial and fungal community. Results showed that the ruminal fluid pH of YC2 and YC4 groups was higher (*P* < 0.05) than that of CON group. Compared with CON group, the microbial protein, propionate and butyrate concentrations and cumulative gas production at 48 h of YC2 group were significantly increased (*P* < 0.05), whereas an opposite trend of ammonia nitrogen and lactate was observed between two groups. Microbial analysis showed that the Chao1 and Shannon indexes of YC2 group were higher (*P* < 0.05) than those of CON group. Additionally, YC supplementation significantly decreased (*P* < 0.05) *Succinivibrionaceae_UCG-001*, *Streptococcus bovis* and *Neosetophoma* relative abundances. An opposite tendency of *Aspergillus* abundance was found between CON and YC treatments. Compared with CON group, the relative abundances of *Prevotella*, *Succiniclasticum*, *Butyrivibrio* and *Megasphaera elsdenii* were significantly increased (*P* < 0.05) in YC2 group, while *Apiotrichum* and *unclassified Clostridiales* relative abundances were decreased (*P* < 0.05). In conclusion, high concentrate substrate supplemented with appropriate YC (1%) can improve ruminal fermentation and regulate bacterial and fungal composition.

**Supplementary Information:**

The online version contains supplementary material available at 10.1186/s13568-024-01692-6.

## Introduction

In recent years, the increased consumption in meat has greatly promoted the development of animal farming, especially in sheep. As a food source that can supply high quality fat and protein as well as key vitamins, mutton plays an important role in the global nutrition security (Vahmani et al. 2020). With the continuous development of intensive meat sheep breeding, fattening has gradually developed into the main mode of meat sheep production. However, the roughage resources with high quality are limited in some parts of the world. In order to meet the nutrient requirements for high production performance, sheep are usually fed with rations high in concentrates. Nevertheless, sheep fed concentrate-rich diets leads to metabolic acidosis, reducing feed efficiency and damaging gastrointestinal function (Lu et al. 2023). Subacute ruminal acidosis (SARA) is a common kind of metabolic acidosis, which can decrease ruminal pH and induce the microbial death to release endotoxins and gastrointestinal dysfunction, leading to reduced feed intake, immune function and production performance of ruminants (Ma et al. 2021). Previous studies have found that buffering agent (Erdman 1988), monensin (Pacheco et al. 2023), lactic acid bacteria (Fenta et al. 2023) and organic acids (Vyas et al. 2015) can be used in alleviating the damage of gastrointestinal function caused by SARA. Although these strategies can attenuate the economic losses caused by SARA to a certain extent, they also have some drawbacks, such as high cost, drug residue and resistance, and cannot fundamentally solve the problem of SARA. Thus, finding an effective method without side effects to prevent the occurrence of SARA is important for intensive breeding industry of meat sheep.

As a kind of microbial fermentation product, yeast culture (YC) is commonly used as a feed additive in animals’ production. YC is mainly composed of yeast extracellular metabolites, variable culture medium after fermentation, cell wall components and a small number of inactive yeast cells (Wang et al. 2023). In addition, YC is rich in nutrients, including amino acids, vitamins, oligosaccharides and unidentified growth factors, which are conducive to promoting production performance and enhancing gastrointestinal function of animals (Wang et al. 2022a). YC can provide essential nutrients mentioned above for lactic acid bacteria, such as *Megasphaera elsdenii* and *Selenomonas ruminantium*, which stimulate the growth of lactate-utilizing bacteria, thereby converting excessive lactic acid into volatile fatty acid (VFA), reducing the accumulation of lactic acid in the rumen and stabilizing the ruminal pH (Gao and Geng 2022). A previous study in dairy cows has verified that YC can regulate rumen pH, maintain the stability of ruminal environment, promote the absorption of VFA by ruminal epithelium and reduce the inflammatory response induced by high concentrate feeding (Li et al. 2016). Moreover, the metabolites of yeast can promote growth of protein-synthesizing (e.g. *Amylococci*, *Amylosarcinae* and *Amylospirillae*) and cellulolytic bacteria (e.g. *Ruminococcus flavefaciens*, *Fibrobacter succinogenes* and *Butyrivibrio fibrisolvens*), thus increasing efficiency of microorganisms using ammonia nitrogen (NH_3_-N) to synthesize the microbial protein (MCP), which finally promote ruminal fermentation and improve feed conversion rate (Halfen et al. 2021).

The influence of yeast products supplementation in the ration on ruminants have been widely investigated. However, the research results exist differences (Zaworski et al. 2014; Olagaray et al. 2019; Dai et al. 2023). The differences of research results might be associated with differences in supplemental levels of yeast products, growth stage of animals and type of strain. Nevertheless, the information of effects of different doses of YC supplementation on ruminal fermentation profile in fattening sheep fed high concentrate diet remains scarce. With the development of science and technology, the in vitro fermentation technique has been commonly utilized as an effective method to evaluate the nutritional value of feed additive of ruminal fermentation (Tunkala et al. 2023). On the other hand, because of the conventional viewpoint in the bacteria as the primary functional microorganisms of rumen, the majority of studies focused on the bacterial composition in the rumen of ruminants (Ma et al. 2020; Zhang et al. 2023). In fact, the fungal community acts as an essential role in the digestion of cellulose (Tong et al. 2023). Therefore, in the current study, we performed an in vitro experiment to assess the influence of YC supplementation levels on ruminal fermentation profile and bacterial and fungal community of high concentrate diet in sheep. We hypothesized that sheep fed high concentrate diet supplemented with appropriate YC dose would regulate the bacterial and fungal composition to alter the ruminal characteristics.

## Materials and methods

### Ruminal fluid preparation

A total of 6 male Hu sheep (32.2 ± 0.8 kg of body weight and 4-month-old) fitted with rumen cannulas were used as the donors of ruminal fluid for in vitro fermentation. Sheep were fed twice daily at 08:00 and 18:00 respectively, and had free access to clean water. The feed ingredients and nutrient composition of experimental diet are presented in Table 1. Before morning feeding, approximately 200 mL of ruminal fluid samples were obtained by ruminal cannulas from each sheep. Subsequently, the samples were filtered by 4 layers of cheesecloth, and then transferred to a prewarmed thermos bottle that was filled with CO_2_ in advance at 39 ℃ in a water bath for subsequent experiment.

**Table 1:** Feed ingredients and nutritional levels of experimental diet (DM basis)

Ingredients, %		Nutrient levels	
Peanut vine	30.00	ME^2^ (MJ/kg)	10.42
Corn	44.80	CP (%)	13.20
Corn germ meal	2.90	NDF (%)	30.35
Corn gluten feed	6.65	ADF (%)	14.45
Wheat bran	2.58	Ca (%)	0.90
Wheat middling	2.80	P (%)	0.48
Barley husk	3.50		
Soybean meal	2.45		
Beet pulp	1.10		
Palm meal	1.40		
Limestone	0.42		
Premix^1^	1.40		

DM, dry matter; CP, crude protein; NDF, neutral detergent fiber; ADF, acid detergent fiber; ME, metabolic energy

^1^ The premix provided following per kilogram of diet: VA 350 000 IU, VD 110 000 IU, VE 3 000 IU, Fe 270 mg, Zn 190 mg, Mn 180 mg, Cu 70 mg, I 16 mg, Se 11 mg, Co 17 mg

^2^ ME was a calculated value, and other nutrient values were measured in the laboratory

### Experimental design and in vitro fermentation inoculation

A total of 5 kg of experimental diet, the same as what was offered to the donor sheep, were collected for utilization as substrate for in vitro fermentation trial. The YC (Angel yeast Co., Ltd., Yichang, Hubei, China; main ingredients: crude protein ≥ 30%; mannan oligosaccharide ≥ 2%; yeast count ≥ 1 × 10^7^ CFU/g) was mixed with the substrate to obtain 5 groups: control with no YC (CON), YC1 (YC proportion was 0.50%), YC2 (1%), YC3 (1.5%) and YC4 (2%). The buffer solution was prepared according to the procedures described by Menke et al. (1979). Next, CO_2_ was pumped into the buffer solution until the pH was 6.8. The in vitro incubation trials were carried out with an anaerobic glass bottle (120 mL). To each individual bottle, 25 mL of filtered ruminal fluid, 500 mg of substrate and 50 mL of buffer solution were added. Incubation bottles were injected with CO_2_ continuously to keep the anaerobic environment. After mixture, the incubation bottles were firmly sealed using rubber plugs and aluminium covers, and subsequently, the bottles were individually connected to the gas inlets of Automated Trace Gas Recording System (AGRS, Beijing, China) (Bai et al. 2018) using medical plastic infusion pipes for continuously recording cumulative gas production (GP). The bottles were cultivated in a constant incubator (Sheyan Instrument Co., Ltd., Shanghai, China) at 39 ℃ for 48 h, and each treatment included 4 replicates.

### Samples collection, analysis and calculation

After incubation, the bottles were separated from AGRS system. Immediately, the pH value of ruminal liquid was determined with a pH meter (INESA Scientific Instruments Co., Ltd., Shanghai, China). Then, the materials of bottles after fermentation were filtered by dried nylon bags (8 × 12 cm with a pore size of 50 μm) to collect incubation fluid. Next, the incubation fluid was placed into 5 mL sterile tubes and stored at -80 ℃ for measurement of microbial composition and fermentation characteristics. Finally, the residues of each incubation bottle were dried at 65 ℃ to a constant weight for measurement of dry matter (DM). The in vitro dry matter digestibility (IVDMD) was calculated from the DM loss following the method described by Zhang and Yang (2011).

The concentration of NH_3_-N in incubation fluid was determined according to the phenol-sodium hypochlorite colorimetry procedures (Broderick and Kang 1980). Moreover, MCP content was analyzed following the method described by Makkar et al. (1982). The gas chromatography (JC-7890, Juchuang Group Co., Ltd., Qingdao, Shandong, China) was utilized to analyze the VFA concentrations, including acetate, propionate, butyrate, isovalerate and valerate (Sun et al. 2022). The non-glucogenic-to-glucogenic acids (NGR) ratio was calculated by NGR = (acetate + 2 butyrate + valerate) / (propionate + valerate) (Zhang and Yang 2011). In this equation, acetate, butyrate, propionate, and valerate are expressed in mole fractions (mol, %) of total VFA yield at the end of incubation.

The cumulative GP at 48 h (GP_48_, mL/g) recorded by AGRS system was used to analyze kinetic indexes of GP using a nonlinear model (Groot et al. 1996) of SAS software (version 9.4; SAS Institute, Inc., Cary, NC, USA) as follows: GP48 = A / [1 + (C/48)^B^ ]. In addition, the average gas production rate (AGPR) was calculated by a formula as follows: AGPR = (A × B) / (4 × C). In the above-mentioned equations, “A” represents theoretical maximum of GP; “B” is an index reflecting the shape of curve; “C” is the time (h) when the maximum GP reaches half.

### DNA extraction and sequencing

Total microbial genomic DNA was isolated from incubation fluid using the Omega DNA kit (Omega Bio-Tek, Norcross, GA, USA) following the manufacturer’s protocols. The concentration and purity of extracted DNA were assessed through a NanoDrop 2000 spectrophotometer (Thermo Scientific, Waltham, MA, USA) and 1.0% agarose gel electrophoresis. To amplify hypervariable region V3-V4 of the bacterial 16 S rRNA gene, the universal primers 338 F (5′-ACTCCTACGGGAGGCAGCAG-3′) and 806R (5′- GGACTACHVGGGTWTCTAAT-3′) were used (Yang et al. 2022b). In addition, the primers ITS1F (5′-CTTGGTCATTTAGAGGAAGTAA-3′) and ITS2R (5′-GCTGCGTTCTTCATCGATGC-3′) of fungi were utilized to amplify the ITS1 of internal transcribed spacer (ITS) region (Huang et al. 2023). The PCR amplification cycling conditions were conducted according to the following process: initial denaturation at 94 °C for 3 min, followed by 30 cycles of denaturing at 94 ℃ for 30 s, annealing at 56 ℃ for 45 s, extension at 72 ℃ for 45 s, and single extension at 72 ℃ for 10 min. Three replicates were carried out for each sample, and PCR products were purified from 2% agarose gel using the kit AxyPrep DNA Gel Extraction Kit (Axygen Biosciences, Union City, CA, USA) and quantified with Quantus™ Fluorometer (Promega, Madison, WI, USA). Sequencing libraries were generated using TruSeq DNA PCR-Free Sample Prep Kit (Illumina, Inc., San Diego, CA, USA) and the library was sequenced on an Illumina HiSeq 2500 platform.

### Bioinformatic analysis

The original sequence of each sample was spliced according to the unique barcodes following by truncation of barcodes sequence. Then, the raw tags were filtered using Trimmomatic (version 0.33) to obtain cleaned tag data (Bolger et al. 2014). Following that, the Uchime algorithm was utilized to eliminate the chimeric sequences using the Gold database to provide clean reads (Edgar et al. 2011). Tags were grouped into operational taxonomic units (OTUs) at 97% identity threshold by the Usearch software (version 10.0) (Edgar 2013). The Silva database (version 138.1) (Quast et al. 2013) and UCLUST (Edgar 2010) were used to assign the taxonomic OTU. Finally, the PyNAST was utilized to compare and filter the representative sequences (Gregory et al. 2010). To avoid affecting community diversity caused by sequencing depth, the samples that have the least number of reads were deemed to be a standard for resampling.

The sequencing data analysis was performed using R software (version 3.5.3). Alpha- and beta-diversity were finished by Vegan. The principal coordinates analysis (PCoA) was carried out through the ape package based on Bray-Curtis dissimilarity matrices obtained by vegdist function of Vegan (Paradis et al. 2004). Moreover, using the adonis function of Vegan, the permutational multivariate analysis of variance (PerMANOVA) was calculated. The heat map was obtained with the dominant bacteria or fungi using the z-score normalization for each sample (z score = [actual relative abundance of a genus − mean relative abundance of the same genus] / standard deviation) (Ma et al. 2020). The heat map was drawn using online resources ImageGP (https://www.bic.ac.cn/ImageGP).

### Statistical analysis

Before analysis, all data were firstly performed normality and homogeneity of variances tests. Variables of DM in vitro degradability, fermentation parameters and in vitro gas production and kinetic parameters were analyzed by one-way ANOVA procedure of the SPSS software (version 22.0). Tukey test was utilized to evaluate the differences among all treatments. The microbial data including alpha-diversity index and relative abundance was analyzed using the non-parametric test of the SPSS software. Orthogonal polynomial contrasts were conducted to assess the linear and quadratic effects of YC levels. Results were presented as means and standard error of the mean. The significance level of DM in vitro degradability, fermentation parameters, in vitro gas production and kinetic parameters and alpha-diversity indexes was declared at *P* < 0.05, and 0.05 ≤ *P* < 0.10 represented a tendency. *P*-values of < 0.05 after false discovery rate correction of microbial composition using the Benjamini–Hochberg procedure for the multiple comparisons were deemed to be significant, and a trend was declared at 0.05 ≤ *P* < 0.10.

## Results

### In vitro degradability of dry matter and ruminal fermentation characteristics

As shown in Table 2, no significant difference (*P* > 0.05) of IVDMD was found among all groups. The in vitro ruminal fluid pH of YC2 and YC4 groups was higher (*P* < 0.05) than that of CON group. Compared with YC2 and YC3 groups, the NH_3_-N concentration in CON and YC4 groups was significantly increased (*P* < 0.05). The MCP content in YC1 and YC2 groups was significantly elevated (*P* < 0.05) when compared to other groups. On the contrary, the lactate concentration of CON group was higher (*P* < 0.05) than that of YC2, YC3 and YC4 groups. In vitro ruminal fluid total VFA, acetate, isovalerate and valerate occurred at similar levels (*P* > 0.05) among all 5 groups. However, compared with CON group, the propionate and butyrate contents of YC2 and YC3 group were significantly increased (*P* < 0.05). Additionally, the acetate-to-propionate ratio and NGR in YC2 and YC3 groups were significantly reduced (*P* < 0.05) as compared with CON, YC1 and YC4 groups.

**Table 2:** Effects of high concentrate diet supplemented with different YC levels on in vitro degradability of DM and ruminal fermentation profile after 48 h of incubation

Items	Treatments					SEM	*P*-value		
	CON	YC1	YC2	YC3	YC4		Treatment	Linear	Quadratic
IVDMD (%)	69.42	69.64	70.83	72.31	73.46	2.043	0.773	0.309	0.830
pH	5.54^b^	5.94^ab^	6.25^a^	6.03^ab^	6.14^a^	0.086	0.022	0.041	0.015
NH_3_-N (mg/dL)	33.71^a^	31.84^ab^	28.67^b^	27.54^b^	34.78^a^	0.879	0.035	0.039	0.021
MCP (mg/mL)	1.87^c^	2.69^a^	2.54^a^	2.04^bc^	2.21^b^	0.008	0.005	0.026	0.010
Lactate (mmol/L)	1.56^a^	1.23^ab^	0.88^b^	0.85^b^	0.92^b^	0.052	0.017	0.022	0.011
Total VFA (mmol/L)	116.43	126.54	119.43	120.86	119.68	4.569	0.011	0.009	0.027
Acetate (mmol/L)	72.95	76.43	67.84	67.94	69.75	2.837	0.031	0.039	0.014
Propionate (mmol/L)	21.77^b^	28.37^ab^	29.53^a^	30.29^a^	28.12^ab^	0.763	0.043	0.078	0.038
Butyrate (mmol/L)	13.28^b^	14.05^ab^	15.56^a^	15.83^a^	14.65^ab^	0.401	0.026	0.061	0.015
Isovalerate (mmol/L)	4.88	4.43	3.79	4.11	4.32	0.340	0.266	0.548	0.380
Valerate (mmol/L)	3.55	3.26	2.71	2.69	2.84	0.123	0.304	0.485	0.255
AP	3.35^a^	2.69^b^	2.30^c^	2.24^c^	2.48^b^	0.003	0.026	0.039	0.018
NGR	4.07^a^	3.41^b^	3.06^c^	3.10^c^	3.29^b^	0.002	0.029	0.040	0.007

DM, dry matter; YC, yeast culture; IVDMD, in vitro dry matter digestibility; NH_3_-N, ammonia nitrogen; MCP, microbial protein; VFA, volatile fatty acid; SEM, standard error of the mean. CON, fermentation substrate with no YC; YC1, fermentation substrate supplemented with 0.50% YC; YC2, 1%; YC3, 1.5%; YC4, 2%. AP = acetate / propionate. NGR = (acetate + 2 butyrate + valerate) / (propionate + valerate). In the same row, values with different small letters differ significantly (*P* < 0.05)

### In vitro ruminal gas production characteristics

Obviously, GP_48_ in CON and YC1 groups was lower (*P* < 0.05) than that in YC2, YC3 and YC4 groups (Table 3). A similar trend of inflection point on the curve parameter was observed. Fermentation substrate supplemented with YC significantly enhanced (*P* < 0.05) theoretical maximum of GP, while an opposite tendency was found in parameter “C” between CON and YC treatments. Besides, the AGPR of YC2 and YC4 groups was significantly elevated (*P* < 0.05) when compared to that of CON and YC1 groups.

**Table 3:** Effects of high concentrate diet supplemented with different YC levels on in vitro gas production and kinetic parameters

Items	Treatments					SEM	*P*-value		
	CON	YC1	YC2	YC3	YC4		Treatment	Linear	Quadratic
GP_48_ (mL/g)	87.61^c^	103.32^b^	116.25^a^	111.08^a^	117.84^a^	2.868	< 0.001	< 0.001	0.026
A	92.37^b^	105.64^a^	117.87^a^	113.17^a^	119.65^a^	3.032	< 0.001	0.016	0.035
B	1.24^c^	1.36^b^	1.48^a^	1.46^a^	1.48^a^	0.021	0.005	0.022	0.047
C	4.58^a^	2.94^b^	2.68^b^	3.16^b^	2.86^b^	0.179	< 0.001	0.004	0.016
AGPR (mL/h)	6.25^c^	12.22^b^	16.27^a^	13.07^ab^	15.48^a^	1.492	< 0.001	< 0.001	0.028

YC, yeast culture; GP_48_, cumulative gas production at 48 h; A, theoretical maximum of GP; B, inflection point on the curve parameter; C, time (h) when the maximum GP reaches half; AGPR, average gas production rate; SEM, standard error of the mean. CON, fermentation substrate with no YC; YC1, fermentation substrate supplemented with 0.50% YC; YC2, 1%; YC3, 1.5%; YC4, 2%. In the same row, values with different small letters differ significantly (*P* < 0.05)

### Bacterial diversity

In total, 685 492 high-quality sequences were obtained from incubated ruminal fluid samples of 5 groups after filtering, with an average of 34 274 ± 1880 sequences per sample. The average sequencing length was 296 ± 2.25 bp (Supplementary Table S1). The numbers of OTUs were 1739, 1820, 2888, 2905 and 2660 in the CON, YC1, YC2, YC3 and YC4 groups, respectively, based on a 97% sequence similarity. Moreover, the average Good’s coverage was more than 0.99, indicating that a sufficient number of sequences were obtained to reflect the bacterial community of the examined samples.

**Fig. 1:**
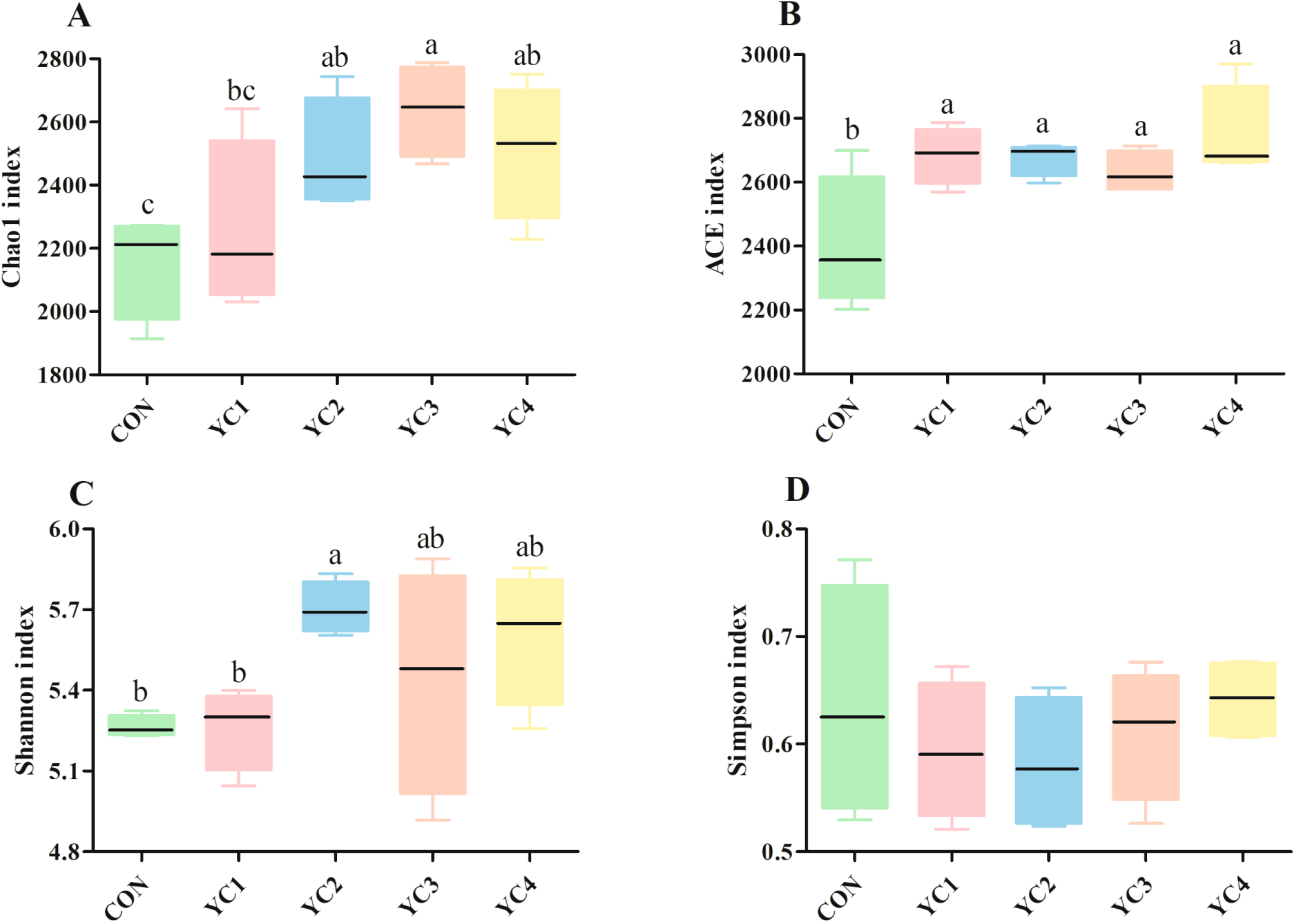
Comparison of the alpha-diversity indexes of bacterial community in incubated ruminal fluid among different treatments. **(A)** Chao1 index; **(B)** ACE index; **(C)** Shannon index; **(D)** Simpson index. YC, yeast culture. CON, fermentation substrate with no YC; YC1, fermentation substrate supplemented with 0.50% YC; YC2, 1%; YC3, 1.5%; YC4, 2%. Columns with different small letters mean significant differences (*P* < 0.05)

**Fig. 2:**
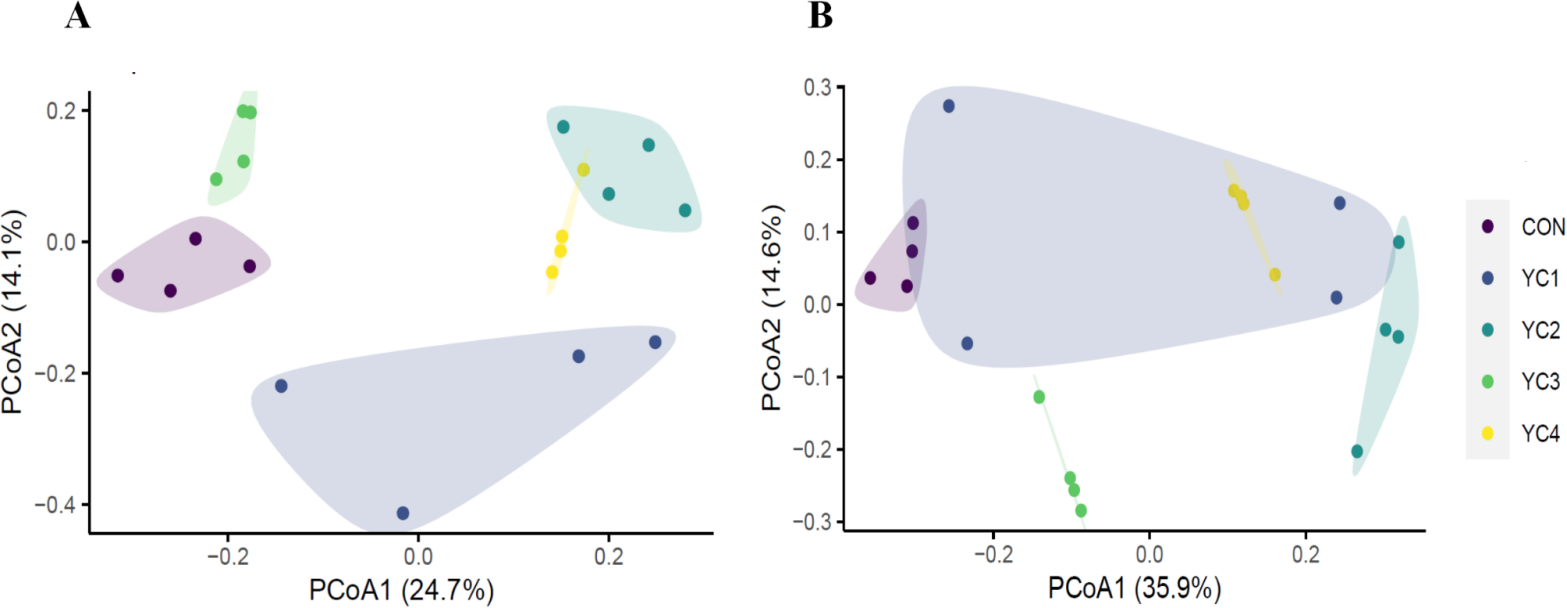
Principal coordinate analysis (PCoA) of bacteria **(A)** and fungal **(B)** communities in the incubated ruminal fluid based on Bray–Curtis distance. YC, yeast culture. CON, fermentation substrate with no YC; YC1, fermentation substrate supplemented with 0.50% YC; YC2, 1%; YC3, 1.5%; YC4, 2%

There was no significant difference (*P* > 0.05) of Simpson index among all groups (Fig. 1D). Nevertheless, the Chao1 index of YC3 group was higher (*P* < 0.05) than that of CON and YC1 groups (Fig. 1A). Likewise, compared with CON and YC1 groups, the Shannon index in YC2 group was significantly increased (*P* < 0.05) (Fig. 1C). Supplementation of YC significantly increased (*P* < 0.05) the ACE index of bacterial community (Fig. 1B). Additionally, PCoA plot based on the Bray-Curtis dissimilarity was used to reflect the differences in bacterial composition of 5 groups. Results showed that the bacterial communities in 5 groups were clearly separated from each other (Fig. 2A).

### Bacterial composition

**Fig. 3:**
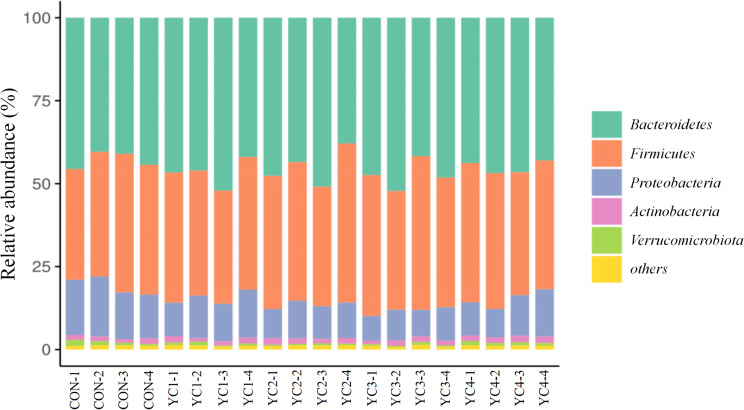
Bacterial composition at phylum level in the incubated ruminal fluid of 5 groups. YC, yeast culture. CON, fermentation substrate with no YC; YC1, fermentation substrate supplemented with 0.50% YC; YC2, 1%; YC3, 1.5%; YC4, 2%

At the taxonomic level, we observed a total of 20 phyla in the incubated ruminal fluid of 20 samples. The *Bacteroidetes* (CON = 42.83%, YC1 = 46.69%, YC2 = 44.99%, YC3 = 47.39% and YC4 = 45.04%), *Firmicutes* (CON = 37.99%, YC1 = 37.77%, YC2 = 41.51%, YC3 = 40.93% and YC4 = 39.72%) and *Proteobacteria* (CON = 15.47%, YC1 = 12.17%, YC2 = 10.13%, YC3 = 8.68% and YC4 = 11.27%) were the dominant bacteria in the incubated ruminal fluid, accounting for more than 95% of the total sequences (Fig. 3 and Supplementary Table S2). No obvious difference (*P* > 0.05) of *Bacteroidetes*, *Firmicutes* and *Actinobacteria* relative abundances was found among all groups. However, compared with CON group, the relative abundance of *Verrucomicrobiota* of YC2 group was significantly reduced (*P* < 0.05). YC supplementation significantly decreased (*P* < 0.05) the relative abundance of *Proteobacteria*. Furthermore, the *Proteobacteria* relative abundance of YC3 group was lower (*P* < 0.05) than that of YC1 group.

**Fig. 4:**
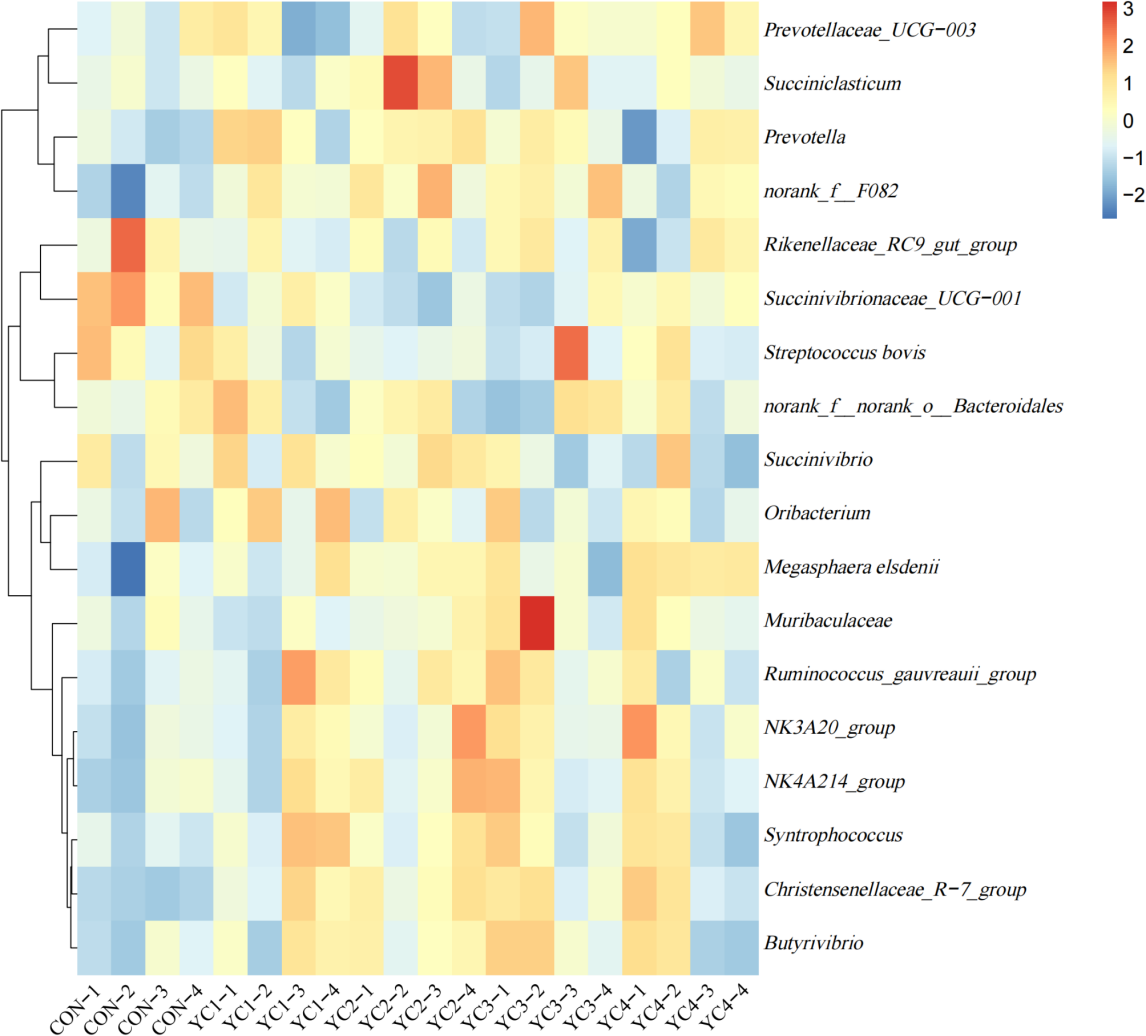
Heat map showing the relative abundance of main bacteria at genus level in the incubated ruminal fluid of 5 groups. The genus with the average relative abundance was ≥ 1% in at least one group. YC, yeast culture. CON, fermentation substrate with no YC; YC1, fermentation substrate supplemented with 0.50% YC; YC2, 1%; YC3, 1.5%; YC4, 2%

At the genus level (Fig. 4 and Supplementary Table S3), the *Succinivibrionaceae_UCG-001* (CON = 27.17%, YC1 = 19.35%, YC2 = 14.42%, YC3 = 16.27% and YC4 = 20.34%) was the most dominant bacterium in the incubated ruminal fluid, followed by *Rikenellaceae_RC9_gut_group* (CON = 17.81%, YC1 = 15.67%, YC2 = 15.54%, YC3 = 17.21% and YC4 = 15.62%) and *Norank_f__F082* (CON = 9.93%, YC1 = 14.27%, YC2 = 15.70%, YC3 = 15.74% and YC4 = 13.23%). The relative abundances of *Succinivibrionaceae_UCG-001* and *Streptococcus bovis* were higher (*P* < 0.05) in CON group than those in YC supplementation groups, whereas an opposite trend of *Norank_f__F082* was found between CON and YC treatments. The YC2 group showed highest relative abundance of *Succiniclasticum* and higher (*P* < 0.05) than other groups. Compared with CON group, the *NK3A20_group*, *Muribaculaceae* and *Megasphaera elsdenii* relative abundances of YC2 and YC4 groups were significantly up-regulated (*P* < 0.05). Besides, the relative abundance of *Prevotellaceae_UCG-003* in YC3 and YC4 groups was higher (*P* < 0.05) than that in CON and YC1 groups. Compared with CON group, the relative abundances of *Prevotella* and *Butyrivibrio* in YC1, YC2 and YC3 groups were significantly up-regulated (*P* < 0.05), while an opposite tendency of *unclassified Clostridiales* was observed between CON and these YC treatments.

### Fungal diversity

For the fungal analysis, 756 134 qualified ITS sequences were detected from incubated ruminal fluid samples, with an average of 37807 ± 1738 sequences per sample, and the average sequencing length was 353 ± 6.38 bp (Supplementary Table S4). All sequences were clustered to 1030, 1052, 1189, 1149 and 1143 fungal OTUs in the CON, YC1, YC2, YC3 and YC4 groups, respectively. In addition, the average Good’s coverage was > 0.99, which indicated that the number of sequences could reflect the diversity and composition of fungal community.

**Fig. 5:**
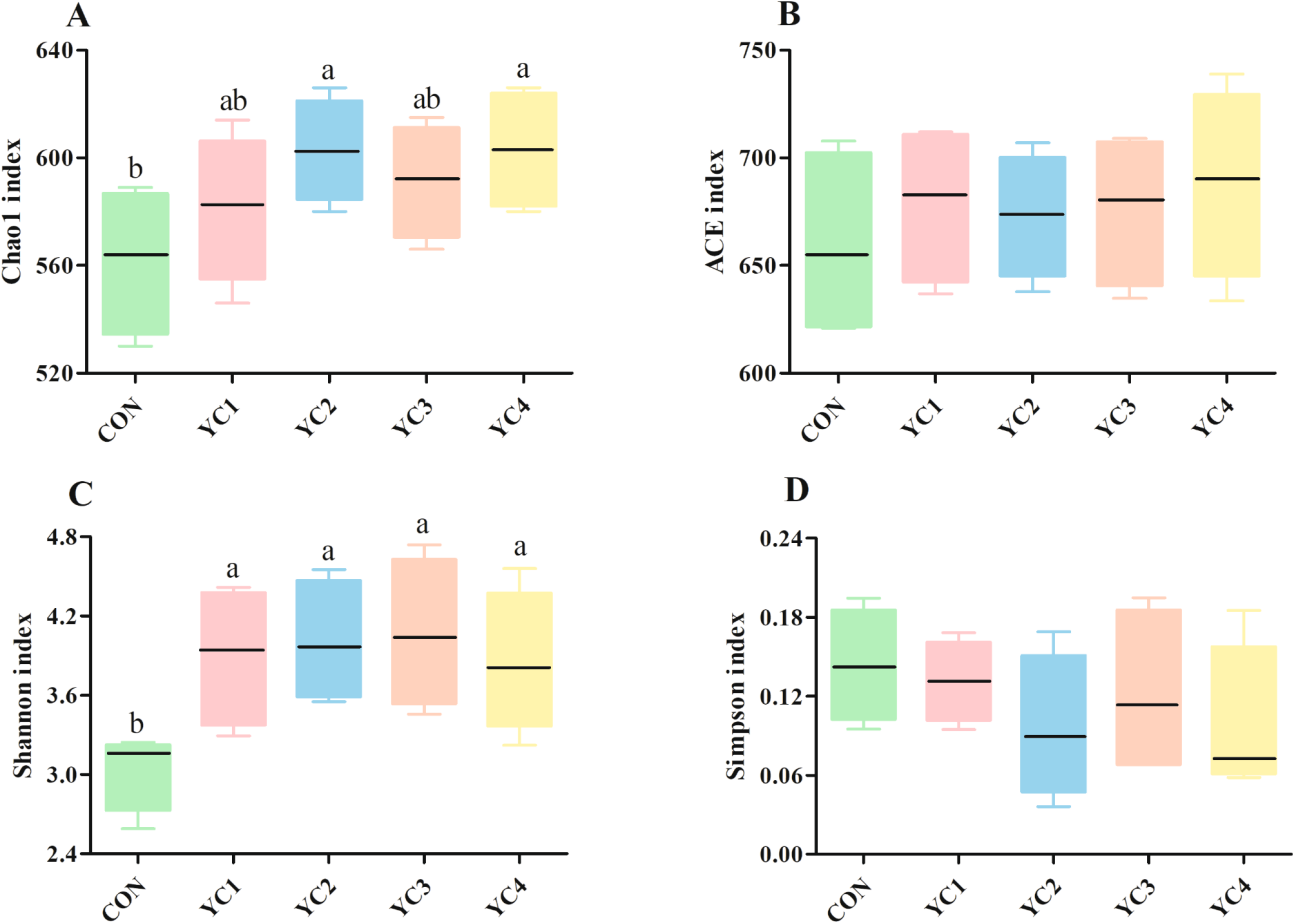
Comparison of the alpha-diversity indexes of fungal community in incubated ruminal fluid among different treatments. **(A)** Chao1 index; **(B)** ACE index; **(C)** Shannon index; **(D)** Simpson index. YC, yeast culture. CON, fermentation substrate with no YC; YC1, fermentation substrate supplemented with 0.50% YC; YC2, 1%; YC3, 1.5%; YC4, 2%. Columns with different small letters mean significant differences (*P* < 0.05)

The ACE (Fig. 5B) and Simpson (Fig. 5D) indexes were similar (*P* < 0.05) among all groups. However, the Chao1 index of YC2 and YC4 groups was higher (*P* < 0.05) than that of CON group (Fig. 5A). Compared with CON group, incubated ruminal fluid supplemented with YC significantly enhanced (*P* < 0.05) the Shannon index (Fig. 5C). Beta-diversity analysis of PCoA displayed obvious differences of fungal community distribution among 5 treatments (Fig. 2B).

### Fungal composition

**Fig. 6:**
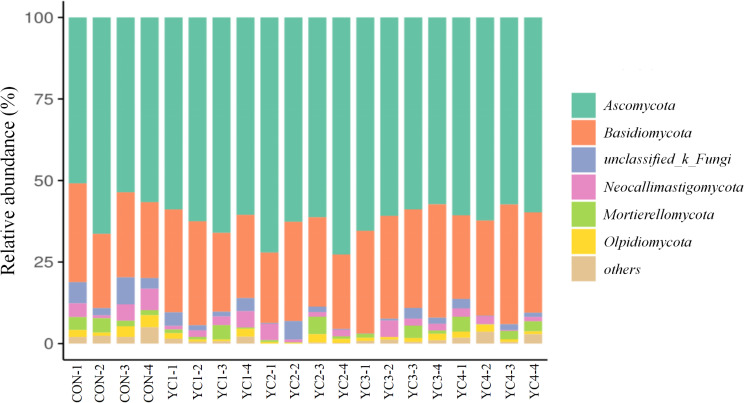
Fungal composition at phylum level in the incubated ruminal fluid of 5 groups. YC, yeast culture. CON, fermentation substrate with no YC; YC1, fermentation substrate supplemented with 0.50% YC; YC2, 1%; YC3, 1.5%; YC4, 2%

The predominant fungal phyla included *Ascomycota* (CON = 56.87%, YC1 = 62.01%, YC2 = 67.17%, YC3 = 60.61% and YC4 = 60.02%) in all incubated ruminal fluid, followed by *Basidiomycota* (CON = 25.61%, YC1 = 28.27%, YC2 = 25.58%, YC3 = 31.99% and YC4 = 30.58%) (Fig. 6 and Supplementary Table S5). Fermentation substrate supplemented with YC did not obviously affect the relative abundances of *Neocallimastigomycota*, *Mortierellomycota* and *Mortierellomycota* (*P* > 0.05). However, the relative abundance of *Ascomycota* in YC2 group was higher (*P* < 0.05) than that in CON group. Compared with CON and YC2 groups, the relative abundance of *Basidiomycota* in YC3 group was significantly elevated (*P* < 0.05). Furthermore, the *unclassified_k__Fungi* relative abundance in CON group was significantly up-regulated (*P* < 0.05) when compared to that in YC3 and YC4 groups.

**Fig. 7:**
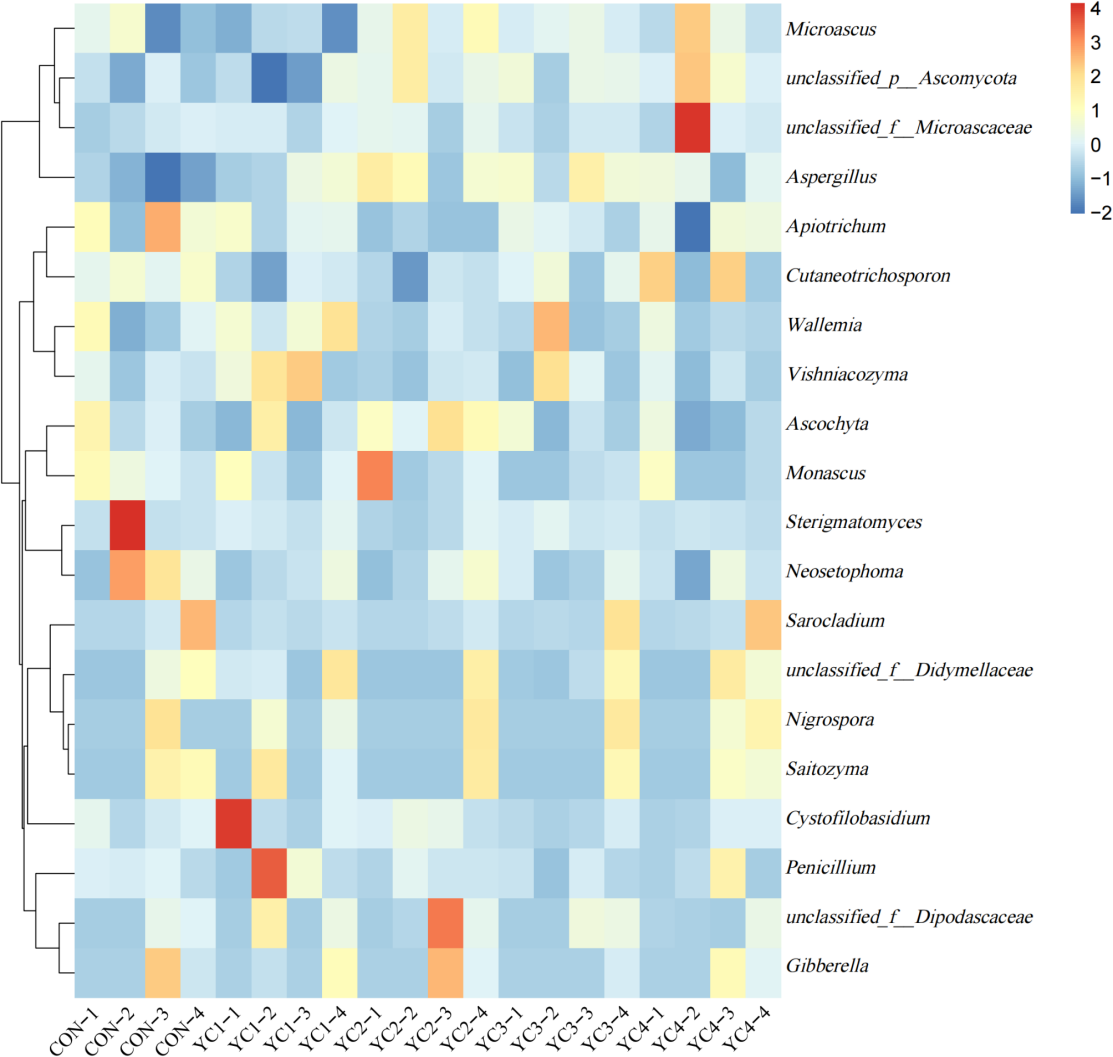
Heat map showing the relative abundance of main fungi at genus level in the incubated ruminal fluid of 5 groups. The genus with the average relative abundance was ≥ 1% in at least one group. YC, yeast culture. CON, fermentation substrate with no YC; YC1, fermentation substrate supplemented with 0.50% YC; YC2, 1%; YC3, 1.5%; YC4, 2%

At the genus level, *Aspergillus* (CON = 16.47%, YC1 = 24.42%, YC2 = 29.48%, YC3 = 28.93% and YC4 = 24.91%), *unclassified_p__Ascomycota* (CON = 8.37%, YC1 = 7.28%, YC2 = 13.04%, YC3 = 11.47% and YC4 = 14.17%) and *Apiotrichum* (CON = 16.59%, YC1 = 13.21%, YC2 = 8.63%, YC3 = 12.06% and YC4 = 11.65%) were the predominant genera (Fig. 7 and Supplementary Table S6). The relative abundance of *Aspergillus* in CON group was higher (*P* < 0.05) than that in YC treatments, whereas an opposite tendency of *Neosetophoma* was found between CON and YC treatments. Compared with CON group, the relative abundances of *Apiotrichum* and *Sterigmatomyces* in YC2 group were significantly decreased (*P* < 0.05). YC2 treatment showed highest relative abundances of *Microascus*, *Ascochyta* and *Monascus*, and higher (*P* < 0.05) than YC1, YC4 and YC3 treatments, respectively. In addition, the *unclassified_p__Ascomycota* relative abundance of YC4 group was significantly elevated (*P* < 0.05) when compared to YC1 group. Conversely, the relative abundance of *Penicillium* in YC1 group was higher (*P* < 0.05) than YC3 group. The relative abundance of *Vishniacozyma* in YC1 group was higher (*P* < 0.05) than that in CON, YC2 and YC4 groups.

## Discussion

After overfeeding of concentrate-rich diets, the excessive fermentable carbohydrates are converted into a large amount of organic acids under the action of ruminal microorganisms, resulting in reduction of ruminal pH and further ruminal acidosis (Ma et al. 2021). YC is rich in B vitamins, trace elements, unknown nutrient factors, oligosaccharides, digestive enzymes, amino acids, organic acids and carbohydrates. In recent years, YC has been widely used in ruminants’ production, which can improve rumen fermentation mode and animal production performance (Gao and Geng 2022; Wang et al. 2022a). Our study investigated the effects of YC on the in vitro ruminal fermentation characteristics and microbial community of high concentrate diet in sheep. Ruminal fluid pH is a comprehensive parameter that can be used to reflect ruminal fermentation and health status. In general, the SARA is characterized by a reduction of ruminal pH below 5.6 (Ma et al. 2021). The lower pH in the rumen has negative influence on the growth and proliferation of microorganisms, and causes the death of ruminal microorganisms to release endotoxins, which leads to dysfunction of the ruminal epithelium (Steele et al. 2011). Our results showed that fermentation substrate supplemented with YC (1% and 2%) increased the ruminal fluid pH, indicating that YC had the ability to improve the reduction of ruminal pH induced by high concentrate. The value of ruminal pH is related to lactate and VFA concentrations. We did not find obvious difference of VFA concentration, but the YC2, YC3 and YC4 groups displayed significantly reduced lactate content, which were in line with pH results. Previous study in dairy cows found that supplementation of YC reduced the ruminal lactate concentration (Dias et al. 2018), which was consistent with our study.

The carbohydrate composition is a key factor to affect the IVDMD (Wang et al. 2022b). In the current study, no difference of IVDMD existed among all groups of incubated ruminal fluid, suggesting that YC could alter the carbohydrate composition of incubated ruminal fluid. However, the NH_3_-N concentration of YC2 and YC3 groups was lower than that of CON and YC4 groups, which indicated that YC could improve the utilization of NH_3_-N in the ruminal fluid. In growing bulls, Zhang et al. (2022) reported that dietary supplementation of YC could decrease the ruminal NH_3_-N content and increase the MCP concentration, which were in accordance with our findings. The positive effect of YC on ruminal NH_3_-N could be attributed with the action of YC on ruminal microbial community. YC can stimulate the microbial growth related to nitrogen utilization, which is manifested in increased utilization of NH_3_-N by ruminal microorganisms (Carpinelli et al. 2021). The decrease in NH_3_-N levels of incubated ruminal fluid was not caused by protein degradation or deamination of protein, but because the stimulation of YC in the ruminal microorganisms to utilize NH_3_-N and synthesize MCP (Carpinelli et al. 2021). Interestingly, the MCP content of YC2 group was significantly increased when compared to CON, YC3 and YC4 groups, which was consistent with NH_3_-N results. Nevertheless, excessive supplementation of YC in fermentation substrate increased NH_3_-N content and reduced MCP content. The possible reason is that some ingredients such as organic acid in YC inhibit the growth of ruminal microorganisms, which lead to reduced utilization of NH_3_-N. But the specific mechanism of action still needs investigation. The microbial community has an important influence on the NH_3_-N utilization and MCP synthesis in the rumen. Thus, we performed the following research to evaluate the effects of YC on bacterial and fungal community of incubated ruminal fluid.

Yeast products are known to regulate the ruminal pH changes, in part by stimulating microorganisms to metabolize lactate into VFA, thereby reducing lactate accumulation in ruminal fluid (Desnoyers et al. 2009). Unfortunately, we only found numerical elevation of total VFA between CON and YC groups. However, the concentrations of propionate and butyrate in incubated ruminal fluid of YC2 and YC3 groups were higher than those of CON group. As an important energy substrate, glucose plays a vital role in animals’ metabolism. For ruminants, liver gluconeogenesis is main process of glucose production, and propionate is an important precursor of gluconeogenesis (Lu et al. 2022). Therefore, although our experiment is in vitro study, the propionate results obtained in the current study could provide more energy for the body. In dairy calves, a previous experiment reported that yeast products increased the proportions of *Butyrivibrio* and lactate-utilizing bacteria, which was beneficial for increasing ruminal propionate and butyrate contents (Xiao et al. 2016). The SARA has adverse effects on the ruminal epithelial function, including induction of inflammatory reaction and reduction absorption ability of VFA and other nutrients (Ma et al. 2021). Butyrate can attenuate the inflammatory reaction in the ruminal epithelium by activating G protein-coupled receptors 41 (Yang et al. 2022a). Moreover, butyrate has the ability to improve the ruminal epithelial morphology and enhance the ruminal villus surface area, which are conducive to increasing the ruminal absorption ability for VFA (Fukumori et al. 2022). Thus, the elevated butyrate concentration in incubated ruminal fluid might have positive influence on relieving VFA accumulation. However, this result need further elucidation using in vivo experiment. In addition, fermentation substrate supplemented with YC significantly reduced acetate-to-propionate ratio, indicating that YC supplementation could promote the transformation of rumen fermentation type to propionate fermentation type under the condition of high concentrate substrate. Ruminal propionate type fermentation can provide more energy, which has positive role for maintaining production performance of animals (Pacheco et al. 2023). We also found that all YC treatments remarkably reduced NGR value, which implied that a shift from acetate towards propionate and butyrate was induced by YC action. In combination with NH_3_-N and MCP results, the appropriate additive amount of YC was from 1 to 1.5%.

The in vitro fermentation GP is a key parameter that can be used to reflect the fermentative degree of substrate nutrients by ruminal microbial community. Generally, higher gas production means higher degree of substrate fermentation (Zhang et al. 2020). A recent study found that yeast products supplementation significantly increased the maximum gas production of fermentation substrate as well as GP_48_ (Cagliari et al. 2023), which were in accordance with our results. The lower gas production in CON group was mainly due to the insufficient activity of microorganisms. After supplementation of YC, the activity of microorganisms in incubated ruminal fluid was enhanced. It might be that YC was rich in minerals, polysaccharides, small peptides and digestive enzymes, which promoted the rapid proliferation of ruminal microorganisms and thus increased the degradation rate of fermentation substrate (Wang et al. 2022a).

As an important digestive organ of ruminants, the rumen harbors a great deal of microorganisms. To a certain degree, the rumen is controllable, and reasonable adjustment can regulate the microbial composition in the rumen, which is helpful for maintaining healthy of animals (Xu et al. 2023). In this experiment, YC supplementation (1%) increased the Chao1, ACE and Shannon indexes of incubated ruminal fluid, indicating that appropriate addition of YC would increase the richness and diversity of ruminal bacterial community. This is inconsistent with a research by Dai et al. (Dai et al. 2023), who found that supplementation with *Saccharomyces cerevisiae* culture did not affect the alpha-diversity of ruminal bacteria. The reason may be that the processing technology of yeast products is different. Consistent with previous study in sheep (Wang et al. 2023), the predominant phyla in the current experiment were *Bacteroidetes*, *Firmicutes* and *Proteobacteria*. The relative abundance of *Proteobacteria* were significantly reduced in YC treatments, which proved that YC can improve the immunity of animals. *Proteobacteria* has been reported to induce gastrointestinal infection or inflammation since it has many pathogenic bacteria including *Escherichia coli* and *Salmonella* (Shin et al. 2015).

At the genus level, YC supplementation significantly reduced the relative abundance of *Streptococcus bovis*, and besides, the YC2 and YC4 groups showed higher relative abundance of *Megasphaera elsdenii* as compared with CON group. The *Megasphaera elsdenii* is lactate-consuming bacteria and *Streptococcus bovis* is lactate-producing bacteria. Our results indicated that YC supplementation could reduce the ruminal lactate content, which was conducive to relieving SARA. The ingredients of YC including amino acid, peptide, vitamin and organic acid can provide nutrients to promote the growth of lactate-consuming bacteria (Desnoyers et al. 2009), thus reducing the accumulation of lactate in the rumen, and finally stabilizing the ruminal pH. We also found that YC supplementation significantly reduced *Succinivibrionaceae_UCG-001* relative abundance. The main fermentation product of *Succinivibrionaceae_UCG-001* is succinic acid, and *Succinivibrionaceae_UCG-001* is a competitor with methanogens for methanogenesis (McCabe et al. 2015). Thus, it is logical to see these genera with reduced abundance in YC treatments because of the elevated gas production. A previous study in dairy cows reported that feeding YC increased the cellulolytic bacteria in the rumen (Halfen et al. 2021). Consistent with previous study, our study found that the relative abundances of *Norank_f__F082*, *Prevotella*, *Succiniclasticum*, *NK3A20_group*, *Muribaculaceae* and *Prevotellaceae_UCG-003* in YC2 group were higher than those in CON group, indicating that YC can improve performance of animals by influencing the ruminal bacterial community to support growth and activity of cellulolytic microorganisms. On the other hand, the main fermentation product of bacteria mentioned-earlier was propionate (Ma et al. 2020). Our results showed that YC supplementation could increase the ruminal propionate content through regulating the bacterial composition. In the rumen, the important butyrate-producing bacteria are *Butyrivibrio fibrisolvens* strains, and the typical characteristic of bacteria belonging to genus *Butyrivibrio* is to produce butyrate (Ma et al. 2022). Improvement in *Butyrivibrio* relative abundance when supplemented with yeast product was reported in previous research (Xiao et al. 2016). Our experiment also found that fermentation substrate supplemented with YC increased *Butyrivibrio* relative abundance, which was beneficial for producing butyrate. Additionally, YC treatments decreased the relative abundance of *unclassified Clostridiales*. In general, *Clostridiales* is less abundant in the rumen, but it can be transformed into pathogenic bacteria under high acid environment (Mahoney-Kurpe et al. 2021), which has negative influence on animal’s health. The mannan oligosaccharide, a ingredient of YC, can inhibit the growth of *Clostridiales* (Wang et al. 2023). Thus, the decreased unclassified *Clostridiales* abundance might improve the production performance of sheep.

In addition to bacterial community, the fungi are also important components of ruminal microorganism. Ruminal fungus can fully degrade plant fiber, because it can penetrate and break the plant cell wall tissue as well as secreting a variety of cellulose degrading enzymes (Yang et al. 2023). Hence, the ruminal fungus can decompose some lignified fibrous materials which cannot be utilized by cellulose-degrading bacteria. Supplementation of YC in fermentation substrate significantly increased the Chao1 and Shannon indexes. The acid environment caused by high concentrate feeding might be unfavorable for growth and reproduction of ruminal fungi. Our finding proved that YC increased the richness and diversity of ruminal fungi. Previously, a study found that high concentrate feeding obviously reduced the relative abundances of *Ascomycota* and *Basidiomycota* in the rumen of dairy cows (Xue et al. 2018). Appropriate supplementation of YC up-regulated the relative abundances of *Ascomycota* and *Basidiomycota* in incubated ruminal fluid, which indicated that the amount of YC consumed could alter the fungal species and YC could attenuate SARA to some extent.

The *Aspergillus* plays an important role in the degradation of cellulose as well as promoting rumen fermentation. In dairy cows suffered from SARA, the relative abundance of *Aspergillus* was reduced (Li 2022). An in vitro experiment found that *Aspergillus oryzae* culture can regulate the ruminal bacterial community to relieve the SARA (Guo et al. 2022). In the current study, YC treatments significantly up-regulated the relative abundance of *Aspergillus*, which could be attributed to that the YC contained oligosaccharide and contributed to the growth of *Aspergillus*. On the contrary, the YC2 group displayed reduced *Apiotrichum* relative abundance when compared to CON group. The *Apiotrichum* has lipolytic activity (Burgstaller et al. 2023), and the decreased *Apiotrichum* relative abundance may protect lipid from degradation in the rumen and can be used in the small intestine. Furthermore, obvious differences of *unclassified_p__Ascomycota*, *Microascus*, *Ascochyta*, *Vishniacozyma*, *Neosetophoma* and *Sterigmatomyces* relative abundances were observed among different treatments. The main function of these fungi is to degrade dietary fiber and produce absorbable compounds for host ruminants (Bhagat et al. 2023). Due to the lower crude fiber in the fermentation substrate, less substrates were supplied for the growth of fungi, thus resulting in the reduced abundance of ruminal fungi in CON group. Nevertheless, these fungal abundances were elevated after YC interventions which suggested that YC supplementation promoted the proliferation and metabolism of fungi in incubated ruminal fluid. YC might accelerate the reaction of pyruvate generating into acetyl-CoA and promote the pyruvate utilization, which produced more ATP to maintain the proliferation of ruminal fungi, but the specific reason requires further investigation. In the future, more studies should be conducted to explore the functions of these microorganisms by metagenomics or culturomics and their response to YC under high concentrate diet condition.

In conclusion, the results from the current study demonstrated that fermentation substrate supplemented with YC (1%) decreased ruminal fluid NH_3_-N and lactate concentrations, whereas increased MCP, propionate and butyrate contents as well as ruminal fluid pH. The gas production of incubated ruminal fluid was increased after YC supplementation. Additionally, YC supplementation regulated the microbial composition, in which the relative abundances of *Butyrivibrio*, *Megasphaera elsdenii*, *Prevotella*, *Succiniclasticum*, *Prevotellaceae_UCG-003* and *Aspergillus* were significantly up-regulated, and the relative abundances of *Succinivibrionaceae_UCG-001*, *Streptococcus bovis*, *unclassified Clostridiales*, *Apiotrichum* and *Neosetophoma* were significantly down-regulated. Therefore, according to our findings, the appropriate supplementation of YC in high concentrate substrate can improve ruminal fermentation and regulate microbial composition of incubated ruminal fluid.

### Electronic supplementary material

Below is the link to the electronic supplementary material.

Supplementary Material 1

## Data Availability

The raw sequencing data from the current study have been deposited in the NCBI database with the accession number PRJNA1054314.
