# Supplementary material for: Effects of yeast culture on in vitro ruminal fermentation and microbial community of high concentrate diet in sheep
Source: AMB Express. 2024 Apr 15;14:37. doi: 10.1186/s13568-024-01692-6 (PMC11018729; doi:10.1186/s13568-024-01692-6)
Supplement: Supplementary file 1 — Supplementary Material 1 [file 13568_2024_1692_MOESM1_ESM.docx]

Supplementary Material

Effects of yeast culture on *in vitro* ruminal fermentation and microbial community of high concentrate diet in sheep

# Supplementary Tables

**Table S1** Bacterial data acquisition of all samples

| **Samples no.** | **Raw sequences** | **Effective sequences** | **Sequencing length** | **OTUs** | **Good’s coverage** |
| --- | --- | --- | --- | --- | --- |
| CON-1 | 34691 | 32105 | 292 | 1785 | 0.992 |
| CON-2 | 32356 | 25644 | 294 | 1689 | 0.993 |
| CON-3 | 34543 | 31320 | 298 | 1625 | 0.992 |
| CON-4 | 37103 | 33075 | 295 | 1856 | 0.994 |
| YC1-1 | 36567 | 31367 | 294 | 1820 | 0.992 |
| YC1-2 | 35639 | 34021 | 296 | 1833 | 0.992 |
| YC1-3 | 32508 | 31356 | 298 | 1921 | 0.993 |
| YC1-4 | 36922 | 34094 | 293 | 1704 | 0.992 |
| YC2-1 | 33021 | 31001 | 296 | 2896 | 0.992 |
| YC2-2 | 36740 | 33065 | 297 | 2888 | 0.991 |
| YC2-3 | 33288 | 30865 | 298 | 2678 | 0.992 |
| YC2-4 | 31859 | 30043 | 298 | 3089 | 0.991 |
| YC3-1 | 32187 | 30912 | 294 | 2765 | 0.991 |
| YC3-2 | 31869 | 30721 | 296 | 2978 | 0.992 |
| YC3-3 | 32389 | 30066 | 295 | 3002 | 0.991 |
| YC3-4 | 34309 | 31084 | 297 | 2876 | 0.993 |
| YC4-1 | 36412 | 33080 | 301 | 2654 | 0.993 |
| YC4-2 | 33806 | 31893 | 298 | 2467 | 0.993 |
| YC4-3 | 36188 | 33043 | 299 | 2563 | 0.991 |
| YC4-4 | 33095 | 30743 | 298 | 2954 | 0.992 |

OTUs, operational taxonomic units; YC, yeast culture. CON, fermentation substrate with no YC; YC1, fermentation substrate supplemented with 0.50% YC; YC2, 1%; YC3, 1.5%; YC4, 2%.

**Table S2** Effects of high concentrate diet supplemented with different YC levels on relative abundance of representative bacteria at phylum level in incubated ruminal fluid

| **Items** | **Treatments** | | | | | **SEM** | ***P*-value** | | |
| --- | --- | --- | --- | --- | --- | --- | --- | --- | --- |
|  | **CON** | **YC1** | **YC2** | **YC3** | **YC4** |  | **Treatment** | **Linear** | **Quadratic** |
| *Bacteroidetes* | 42.83 | 46.69 | 44.99 | 47.39 | 45.04 | 0.862 | 0.539 | 0.424 | 0.276 |
| *Firmicutes* | 37.99 | 37.77 | 41.51 | 40.93 | 39.72 | 0.812 | 0.526 | 0.275 | 0.377 |
| *Proteobacteria* | 15.47^a^ | 12.17^b^ | 10.13^bc^ | 8.68^c^ | 11.27^bc^ | 0.642 | 0.002 | 0.001 | 0.003 |
| *Actinobacteria* | 1.44 | 1.49 | 1.68 | 1.43 | 1.78 | 0.068 | 0.407 | 0.211 | 0.786 |
| *Verrucomicrobiota* | 1.08^a^ | 0.728^ab^ | 0.499^b^ | 0.540^ab^ | 0.987^ab^ | 0.027 | 0.038 | 0.490 | 0.010 |

YC, yeast culture; SEM, standard error of the mean. CON, fermentation substrate with no YC; YC1, fermentation substrate supplemented with 0.50% YC; YC2, 1%; YC3, 1.5%; YC4, 2%. The phylum with the average relative abundance was ≥ 1% in at least one group. In the same row, values with different small letters differ significantly (*P* < 0.05).

**Table S3** Effects of high concentrate diet supplemented with different YC levels on relative abundance of representative bacteria at genus level in incubated ruminal fluid

| **Items** | **Treatments** | | | | | **SEM** | ***P*-value** | | |
| --- | --- | --- | --- | --- | --- | --- | --- | --- | --- |
|  | **CON** | **YC1** | **YC2** | **YC3** | **YC4** |  | **Treatment** | **Linear** | **Quadratic** |
| *Succinivibrionaceae_UCG-001* | 27.17^a^ | 19.35^bc^ | 14.42^c^ | 16.27^bc^ | 20.34^b^ | 1.198 | 0.001 | 0.006 | <0.001 |
| *Rikenellaceae_RC9_gut_group* | 17.81 | 15.67 | 15.54 | 17.21 | 15.62 | 0.520 | 0.566 | 0.463 | 0.563 |
| *Norank_f__F082* | 9.93^b^ | 14.27^a^ | 15.70^a^ | 15.74^a^ | 13.23^a^ | 0.651 | 0.010 | 0.031 | 0.002 |
| *Prevotella* | 4.38^b^ | 7.36^a^ | 7.80^a^ | 6.82^a^ | 5.60^ab^ | 0.486 | 0.042 | 0.149 | 0.018 |
| *Succiniclasticum* | 3.46^b^ | 3.64^b^ | 7.39^a^ | 4.02^b^ | 3.93^b^ | 0.168 | 0.042 | 0.720 | 0.004 |
| *NK3A20_group* | 2.13^b^ | 2.88^ab^ | 3.37^a^ | 3.32^ab^ | 3.55^a^ | 0.107 | 0.025 | 0.065 | 0.042 |
| *Muribaculaceae* | 1.81^b^ | 1.52^b^ | 2.23^a^ | 3.18^a^ | 2.39^a^ | 0.085 | 0.259 | 0.107 | 0.702 |
| *Succinivibrio* | 1.89 | 2.09 | 2.18 | 1.66 | 1.59 | 0.109 | 0.378 | 0.197 | 0.222 |
| *Christensenellaceae_R-7_group* | 1.05 | 2.00 | 2.18 | 2.09 | 1.99 | 0.141 | 0.049 | 0.032 | 0.030 |
| *Streptococcus bovis* | 2.15^a^ | 1.33^b^ | 1.11^b^ | 1.48^b^ | 1.44^b^ | 0.058 | 0.994 | 0.974 | 0.975 |
| *Syntrophococcus* | 1.30 | 1.88 | 1.71 | 1.70 | 1.58 | 0.093 | 0.400 | 0.566 | 0.133 |
| *Butyrivibrio* | 1.18^b^ | 1.67^a^ | 1.74^a^ | 1.91^a^ | 1.54^ab^ | 0.121 | 0.430 | 0.282 | 0.131 |
| *NK4A214_group* | 1.10 | 1.34 | 1.51 | 1.41 | 1.36 | 0.074 | 0.546 | 0.289 | 0.200 |
| *Megasphaera elsdenii* | 1.08^b^ | 1.38^ab^ | 1.48^a^ | 1.31^ab^ | 1.72^a^ | 0.032 | 0.037 | 0.014 | 0.278 |
| *Prevotellaceae_UCG-003* | 1.03^b^ | 0.942^b^ | 1.09^ab^ | 1.22^a^ | 1.37^a^ | 0.019 | 0.042 | 0.156 | 0.024 |
| *Ruminococcus_gauvreauii_group* | 0.881 | 1.23 | 1.26 | 1.32 | 1.04 | 0.074 | 0.432 | 0.256 | 0.567 |
| *unclassified Clostridiales* | 1.12^a^ | 0.895^b^ | 0.789^b^ | 0.882^b^ | 0.923^ab^ | 0.073 | 0.988 | 0.668 | 0.923 |
| *Oribacterium* | 0.752 | 1.15 | 0.760 | 0.770 | 0.761 | 0.094 | 0.646 | 0.608 | 0.621 |

YC, yeast culture; SEM, standard error of the mean. CON, fermentation substrate with no YC; YC1, fermentation substrate supplemented with 0.50% YC; YC2, 1%; YC3, 1.5%; YC4, 2%. The genus with the average relative abundance was ≥ 1% in at least one group. In the same row, values with different small letters differ significantly (*P* < 0.05).

**Table S4** Fungal data acquisition of all samples

| **Samples no.** | **Raw sequences** | **Effective sequences** | **Sequencing length** | **OTUs** | **Good’s coverage** |
| --- | --- | --- | --- | --- | --- |
| CON-1 | 43577 | 40151 | 353 | 1023 | 0.998 |
| CON-2 | 39460 | 35451 | 338 | 987 | 0.983 |
| CON-3 | 40698 | 37218 | 346 | 1043 | 0.995 |
| CON-4 | 38771 | 35572 | 350 | 1066 | 0.996 |
| YC1-1 | 43217 | 39894 | 346 | 994 | 0.998 |
| YC1-2 | 41087 | 37932 | 353 | 1130 | 0.994 |
| YC1-3 | 42370 | 38802 | 344 | 1094 | 0.995 |
| YC1-4 | 38764 | 36272 | 349 | 991 | 0.996 |
| YC2-1 | 45976 | 41376 | 352 | 1132 | 0.990 |
| YC2-2 | 41356 | 37489 | 349 | 1234 | 0.994 |
| YC2-3 | 42931 | 39170 | 362 | 1189 | 0.994 |
| YC2-4 | 38833 | 36116 | 354 | 1201 | 0.992 |
| YC3-1 | 40765 | 37267 | 358 | 1121 | 0.993 |
| YC3-2 | 41321 | 37383 | 357 | 1108 | 0.997 |
| YC3-3 | 41855 | 38419 | 352 | 1204 | 0.996 |
| YC3-4 | 42016 | 37634 | 354 | 1165 | 0.997 |
| YC4-1 | 39875 | 34663 | 357 | 1384 | 0.995 |
| YC4-2 | 44136 | 39958 | 365 | 986 | 0.995 |
| YC4-3 | 41298 | 38068 | 359 | 1104 | 0.989 |
| YC4-4 | 42031 | 37298 | 356 | 1098 | 0.990 |

OTUs, operational taxonomic units; YC, yeast culture. CON, fermentation substrate with no YC; YC1, fermentation substrate supplemented with 0.50% YC; YC2, 1%; YC3, 1.5%; YC4, 2%.

**Table S5** Effects of high concentrate diet supplemented with different YC levels on relative abundance of representative fungi at phylum level in incubated ruminal fluid

| **Items** | **Treatments** | | | | | **SEM** | ***P*-value** | | |
| --- | --- | --- | --- | --- | --- | --- | --- | --- | --- |
|  | **CON** | **YC1** | **YC2** | **YC3** | **YC4** |  | **Treatment** | **Linear** | **Quadratic** |
| *Ascomycota* | 56.87^b^ | 62.01^ab^ | 67.17^a^ | 60.61^ab^ | 60.02^ab^ | 1.205 | 0.028 | 0.516 | 0.018 |
| *Basidiomycota* | 25.61^b^ | 28.27^ab^ | 25.58^b^ | 31.99^a^ | 30.58^ab^ | 0.951 | 0.041 | 0.036 | 0.894 |
| *unclassified_k__Fungi* | 5.06^a^ | 2.81^ab^ | 1.96^ab^ | 1.45^b^ | 1.54^b^ | 0.503 | 0.037 | 0.019 | 0.202 |
| *Neocallimastigomycota* | 4.18 | 2.71 | 2.32 | 2.33 | 1.66 | 0.420 | 0.434 | 0.088 | 0.570 |
| *Mortierellomycota* | 2.89 | 1.68 | 1.71 | 1.50 | 2.57 | 0.393 | 0.781 | 0.785 | 0.237 |
| *Olpidiomycota* | 2.54 | 1.33 | 1.12 | 1.25 | 1.45 | 0.216 | 0.235 | 0.140 | 0.085 |

YC, yeast culture; SEM, standard error of the mean. CON, fermentation substrate with no YC; YC1, fermentation substrate supplemented with 0.50% YC; YC2, 1%; YC3, 1.5%; YC4, 2%. The phylum with the average relative abundance was ≥ 1% in at least one group. In the same row, values with different small letters differ significantly (*P* < 0.05).

**Table S6** Effects of high concentrate diet supplemented with different YC levels on relative abundance of representative fungi at genus level in incubated ruminal fluid

| **Items** | **Treatments** | | | | | **SEM** | ***P*-value** | | |
| --- | --- | --- | --- | --- | --- | --- | --- | --- | --- |
|  | **CON** | **YC1** | **YC2** | **YC3** | **YC4** |  | **Treatment** | **Linear** | **Quadratic** |
| *Aspergillus* | 16.47^b^ | 24.42^a^ | 29.48^a^ | 28.93^a^ | 24.91^a^ | 1.493 | 0.022 | 0.021 | 0.009 |
| *unclassified_p__Ascomycota* | 8.37^ab^ | 7.28^b^ | 13.04^ab^ | 11.47^ab^ | 14.17^a^ | 0.939 | 0.031 | 0.014 | 0.971 |
| *Apiotrichum* | 16.59^a^ | 13.21^ab^ | 8.63^b^ | 12.06^ab^ | 11.65^ab^ | 1.046 | 0.042 | 0.129 | 0.057 |
| *Microascus* | 7.58^ab^ | 5.94^b^ | 11.34^a^ | 9.17^ab^ | 10.40^ab^ | 0.722 | 0.037 | 0.039 | 0.739 |
| *Wallemia* | 5.36 | 9.60 | 4.09 | 6.58 | 4.49 | 0.997 | 0.447 | 0.512 | 0.584 |
| *Ascochyta* | 4.10^ab^ | 3.61^ab^ | 6.13^a^ | 3.33^ab^ | 2.92^b^ | 0.346 | 0.038 | 0.376 | 0.052 |
| *Vishniacozyma* | 2.81^b^ | 5.95^a^ | 2.20^b^ | 3.62^ab^ | 2.32^b^ | 0.374 | 0.043 | 0.059 | 0.424 |
| *Penicillium* | 2.60^ab^ | 4.91^a^ | 2.41^ab^ | 1.78^b^ | 2.76^ab^ | 0.256 | 0.029 | 0.046 | 0.873 |
| *Neosetophoma* | 2.94^a^ | 1.27^b^ | 1.45^b^ | 1.20^b^ | 1.18^b^ | 0.082 | 0.019 | 0.071 | 0.208 |
| *Cutaneotrichosporon* | 2.17 | 1.05 | 0.937 | 1.65 | 2.40 | 0.247 | 0.234 | 0.535 | 0.033 |
| *Sterigmatomyces* | 3.67^a^ | 1.68^ab^ | 0.932^b^ | 1.70^ab^ | 1.17^b^ | 0.312 | 0.045 | 0.094 | 0.321 |
| *Monascus* | 2.66^ab^ | 1.87^ab^ | 2.94^a^ | 0.564^b^ | 1.18^ab^ | 0.422 | 0.046 | 0.252 | 0.884 |
| *unclassified_f__Microascaceae* | 0.75 | 1.07 | 1.40 | 0.80 | 3.18 | 0.467 | 0.483 | 0.189 | 0.433 |
| *unclassified_f__Dipodascaceae* | 0.70 | 1.37 | 2.12 | 0.97 | 0.49 | 0.372 | 0.710 | 0.770 | 0.221 |
| *Cystofilobasidium* | 1.14 | 2.96 | 1.55 | 0.442 | 0.703 | 0.473 | 0.518 | 0.336 | 0.495 |
| *unclassified_f__Didymellaceae* | 0.644 | 1.055 | 0.556 | 0.451 | 0.549 | 0.123 | 0.972 | 0.970 | 0.725 |
| *Saitozyma* | 1.062 | 0.524 | 0.391 | 0.323 | 0.481 | 0.142 | 0.965 | 0.614 | 0.620 |
| *Gibberella* | 0.741 | 0.452 | 1.041 | 0.124 | 0.563 | 0.198 | 0.836 | 0.657 | 0.848 |
| *Sarocladium* | 5.58 | 0.78 | 0.77 | 4.08 | 4.99 | 1.457 | 0.773 | 0.849 | 0.273 |
| *Nigrospora* | 1.012 | 0.451 | 0.648 | 0.541 | 0.654 | 0.129 | 0.996 | 0.809 | 0.775 |

YC, yeast culture; SEM, standard error of the mean. CON, fermentation substrate with no YC; YC1, fermentation substrate supplemented with 0.50% YC; YC2, 1%; YC3, 1.5%; YC4, 2%. The genus with the average relative abundance was ≥ 1% in at least one group. In the same row, values with different small letters differ significantly (*P* < 0.05).
